# Large-Scale Fusion of Gray Matter and Resting-State Functional MRI Reveals Common and Distinct Biological Markers across the Psychosis Spectrum in the B-SNIP Cohort

**DOI:** 10.3389/fpsyt.2015.00174

**Published:** 2015-12-21

**Authors:** Zheng Wang, Shashwath A. Meda, Matcheri S. Keshavan, Carol A. Tamminga, John A. Sweeney, Brett A. Clementz, David J. Schretlen, Vince D. Calhoun, Su Lui, Godfrey D. Pearlson

**Affiliations:** ^1^Mental Health Institute of the Second Xiangya Hospital, Central South University, Changsha, China; ^2^Olin Neuropsychiatry Research Center, Institute of Living at Hartford Hospital, Hartford, CT, USA; ^3^Department of Psychiatry, Beth Israel Deaconess Hospital, Harvard Medical School, Boston, MA, USA; ^4^Department of Psychiatry, University of Texas Southwestern Medical Center, Dallas, TX, USA; ^5^Department of Psychology, University of Georgia, Athens, GA, USA; ^6^Department of Psychiatry, Johns Hopkins University, Baltimore, MD, USA; ^7^The Mind Research Network, Albuquerque, NM, USA; ^8^Department of Psychiatry, Yale University, New Haven, CT, USA; ^9^Department of Radiology, Huaxi MR Research Center, West China Hospital of Sichuan University, Chengdu, China

**Keywords:** schizophrenia, schizoaffective, bipolar, relatives, multimodal neuroimaging, joint-independent component analysis

## Abstract

To investigate whether aberrant interactions between brain structure and function present similarly or differently across probands with psychotic illnesses [schizophrenia (SZ), schizoaffective disorder (SAD), and bipolar I disorder with psychosis (BP)] and whether these deficits are shared with their first-degree non-psychotic relatives. A total of 1199 subjects were assessed, including 220 SZ, 147 SAD, 180 psychotic BP, 150 first-degree relatives of SZ, 126 SAD relatives, 134 BP relatives, and 242 healthy controls (1). All subjects underwent structural MRI (sMRI) and resting-state functional MRI (rs-fMRI) scanning. Joint-independent component analysis (jICA) was used to fuse sMRI gray matter and rs-fMRI amplitude of low-frequency fluctuations data to identify the relationship between the two modalities. jICA revealed two significantly fused components. The association between functional brain alteration in a prefrontal–striatal–thalamic–cerebellar network and structural abnormalities in the default mode network was found to be common across psychotic diagnoses and correlated with cognitive function, social function, and schizo-bipolar scale scores. The fused alteration in the temporal lobe was unique to SZ and SAD. The above effects were not seen in any relative group (including those with cluster-A personality). Using a multivariate-fused approach involving two widely used imaging markers, we demonstrate both shared and distinct biological traits across the psychosis spectrum. Furthermore, our results suggest that the above traits are psychosis biomarkers rather than endophenotypes.

## Introduction

Whether schizophrenia (SZ), schizoaffective disorder (SAD), and psychotic bipolar disorder are distinct illnesses or represent a continuum continues to be debated (2–4). There is overlap among clinical symptoms (5), cognitive functional deficits (6), and disease risk genes (7) among these disorders (8) that challenge traditional diagnostic categories. Clarifying similarities and differences in anatomical and functional deficits among psychotic probands may contribute to the better understanding of the mechanisms underlying psychotic disorders. The above disorders are highly heritable (9, 10), and illness-related genes associated with the abnormalities in brain structure and function (11) may also be present in their unaffected relatives. Thus, shared abnormalities between probands and unaffected relatives can serve as endophenotypes, which may provide biological genetic substrates for improved diagnostic classification (12), and ultimately may lead to better, more focused treatments.

Psychotic illnesses are widely assumed to be brain disorders characterized by distributed cerebral dysconnectivity across large-scale neural networks. Resting-state networks reflect correlated spontaneous fluctuations in brain regional activity at rest (13) and show a similar correspondence to task-related networks that control brain action and cognition (14). A recent resting-state functional MRI (rs-fMRI) study reported that SZ, SAD, and psychotic bipolar disorder share disruptions within the fronto-parietal control network (7). Both default mode network (DMN) and prefrontal–thalamic–cerebellar network connectivity have been reported to be abnormal in probands with SZ and bipolar disorder (15, 16) and between probands and relatives with SZ (17–19). Lui et al. (20) compared resting-state functional network connectivity between SZ and psychotic bipolar probands and their unaffected first-degree relatives and found probands with SZ and psychotic bipolar shared deficits in striatal–thalamic–cortical network as well as bipolar relatives. Independent structural MRI (sMRI) studies reveal both common and unique regional gray matter (GM) abnormalities across psychotic probands (21–24). Ivleva et al. (24) reported that SZ, SAD, psychotic bipolar disorder probands, and their relatives with psychosis showed overlapping GM deficits throughout the neocortex as a psychosis endophenotype. Collectively, overlaps and differences in both functional and structural networks across psychosis probands and their relatives have been identified, but no studies to date have examined both structural and functional deficits together. Brain regions are highly interconnected and local changes in brain structure may result in altered brain activity in distant regions (25, 26). Inter-regional correlations of GM volume may reflect changed inter-regional functional connectivity (27). Thus, examining abnormalities in structure and function together may help better characterize illness-related features and provide more information than each measure independently.

Joint-independent component analysis (jICA), a data-driven feature-based approach, enables joint analysis of different data types, for example, relationships between brain function and structure (28). In the present study, we utilized two commonly employed techniques to quantify anatomy and function. Anatomical data were indexed by GM volumes obtained through voxel-based morphometry (VBM), while resting-state function was measured using amplitude of low-frequency fluctuations (ALFF) (0.01–0.08 Hz) of the blood oxygen level dependent. ALFF measures, particularly in the 0.01–0.08 Hz frequency range have been demonstrated to be physiologically relevant and related to neuronal fluctuations in brain GM in resting state (29, 30). In the current study, we utilized the joint approach to integrate GM and ALFF, to investigate joint structure–function anomalies across the SZ-psychotic bipolar disorder spectrum using data from the large-scale multi-site bipolar-schizophrenia network on intermediate phenotypes (B-SNIP) psychosis study (31). Our aims were to (1) detect whether aberrancies detected by fusing rs-fMRI and sMRI would be specific to SZ, SAD, or bipolar I disorder with psychosis (BP) or shared by these psychotic disorders relative to healthy controls; (2) investigate whether these abnormalities across two modalities would be shared by probands and their non-psychotic relatives, suggesting that they may represent endophenotypes across the psychosis dimension.

## Materials and Methods

### Participants

A total of 1199 subjects (passing quality control) were used for the current analysis. Subjects were drawn from the B-SNIP study from six sites, including 220 SZ, 180 psychotic BP, 147 SAD, 150 first-degree relatives of SZ, 134 first-degree relatives of psychotic BP, 126 first-degree relatives of SAD, and 242 healthy controls. The details of characteristics of the B-SNIP clinical population are described in Ref. (31). All participants provided written informed consent approved separately by institutional review boards of individual sites after a complete explanation of the study. All probands and relatives were diagnosed using the Structured Clinical Interview for DSM-IV Axis I Disorder, Patient Edition (SCID-I/P) (32). Relatives were also diagnosed with the structured interview for DSM-IV personality (SIDP-IV) (33) for axis-II diagnoses. Relatives meeting the criteria for axis I proband-like psychotic disorders (*N* = 64) were classified to the corresponding proband groups and those with no axis I or no lifetime psychotic diagnoses were included in the non-psychotic relative groups. In addition, relatives without a psychotic disorder were administered the SIDP-IV (34) and were considered to have elevated psychosis spectrum personality traits if meeting full or within one criteria of Cluster-A (psychosis spectrum; *N* = 63) Axis-II diagnosis. Healthy controls were evaluated using the SCID Non-Patient Edition to confirm lifetime absence of Axis I illness or a family history of SZ-bipolar spectrum disorders. All probands were assessed with positive and negative syndrome scale (PANSS) (35), Montgomery–Åsberg depression rating scale (MADRS) (36), Young Mania Rating Scale (YMRS) (37), and schizo-bipolar scale (SBS) (5). Additionally, all subjects were assessed with brief assessment of cognition in schizophrenia (BACS) (38) and the Birchwood social functioning scale (SFS) (39).

The demographic and clinical characteristics of study sample are outlined in Table 1, and details are described in the previous B-SNIP papers (24, 31). Medication data are listed in Table S2 in Supplementary Material.

**Table 1:** **Demographic and clinical characteristics of the study sample**.

Variable^a^	Schizophrenia probands (*n* =220)		Schizoaffective disorder probands (*n* =147)		Psychotic bipolar probands (*n* =180)		Relatives of schizophrenia (*n* =150)		Relatives of schizoaffective disorder (*n* =126)		Relatives of psychotic bipolar (*n* =134)		Healthy controls (*n* =242)		Statistic^c^	

	*N*	%	*N*	%	*N*	%	*N*	%	*N*	%	*N*	%	*N*	%	χ^2^	*p*
**Male gender**	145	65.91	66	44.90	58	32.22	49	32.67	40	31.75	49	36.57	103	42.56	71.27	2 × 10^−13^

	**Mean**	**SD**	**Mean**	**SD**	**Mean**	**SD**	**Mean**	**SD**	**Mean**	**SD**	**Mean**	**SD**	**Mean**	**SD**	***F***	***p***

Age (years)	35.15	12.31	35.08	12.01	36.94	13.04	43.33	15.55	41.01	16.14	40.59	16.13	38.14	12.65	7.72	4 × 10^−8^
**PANSS**																
Positive	16.91	5.42	18.12	5.32	12.77	4.38	–	–	–	–	–	–	–	–	50.47	1 × 10^−22^
Negative	16.27	5.93	15.49	4.95	11.79	3.61	–	–	–	–	–	–	–	–	40.96	3 × 10^−17^
General	32.02	8.72	34.80	9.00	28.60	8.04	–	–	–	–	–	–	–	–	20.57	3 × 10^−9^
Total	65.20	16.78	68.41	16.28	53.17	13.48	–	–	–	–	–	–	–	–	43.74	3 × 10^−18^
MADRS	8.90	7.93	14.31	10.26	10.51	8.99	–	–	–	–	–	–	–	–	15.76	2 × 10^−7^
YMRS	5.76	5.79	7.69	6.48	5.69	6.46	–	–	–	–	–	–	–	–	5.22	0.006
SBS	7.79	1.36	5.01	1.60	1.35	1.24	–	–	–	–	–	–	–	–	1006.21	2 × 10^−178^
**BACS^b^ (*z*)**																
Verbal memory	−1.14	1.32	−1.04	1.39	−0.44	1.28	−0.08	1.09	−0.46	1.26	−0.13	1.07	−0.04	1.08	23.13	8 × 10^−26^
Token motor	−1.33	1.18	−1.36	1.17	−0.95	1.24	−0.33	1.16	−0.24	1.03	−0.31	1.07	0.02	1.12	39.24	3 × 10^−43^
Digit sequencing	−1.26	1.20	−0.94	1.28	−0.51	1.11	−0.38	1.14	-0.27	1.15	−0.03	1.11	−0.06	1.12	26.71	8 × 10^−30^
Verbal fluency	−0.76	1.15	−0.50	1.27	−0.20	1.23	−0.93	1.08	0.02	1.20	0.07	1.07	0.14	1.05	14.20	1 × 10^−15^
Symbol coding	−1.41	1.10	−1.37	1.18	−0.87	1.01	−0.37	1.08	−0.39	1.10	−0.07	1.04	−0.00	1.01	49.42	1 × 10^−53^
Tower of London	−0.87	1.39	−0.70	1.30	−0.28	1.10	−0.19	1.08	−0.18	1.27	0.11	0.85	0.02	1.17	15.80	2 × 10^−17^
Composite score	−1.79	1.34	−1.54	1.37	−0.87	1.24	−0.38	1.19	−0.40	1.25	−0.09	1.11	0.02	1.17	54.99	3 × 10^−59^
SFS	123.27	23.13	119.28	24.42	134.10	22.77	151.03	18.23	144.64	21.89	151.42	21.60	156.09	15.94	68.31	2 × 10^−70^

*^a^PANSS, positive and negative syndrome scale; MRADS, Montgomery–Åsberg depression rating scale; YMRS, Young Mania Rating Scale; SBS, schizo-bipolar scale; BACS, brief assessment of cognition in schizophrenia; SFS, Birchwood social functioning scale*.

*^b^*z*-Scores are calculated using the overall means and standard deviations of all healthy controls and corrected with age and sex*.

*^c^*Post hoc* statistic are presented as follows: age: SADR, SZR > HC; SZR, BPR, SADR > BP; HC, BPR, SADR, SZR > SZ; BPR, SADR, SZR > SAD. PANSS_Positive: SZ > BP; SAD > SZ, BP; PANSS_Negative: SZ > BP, SAD; SAD > BP; PANSS General: SAD > SZ > BP. MADRS: BP > HC; SZ > HC; SAD > all groups. YMRS: BP > HC; SZ > HC; SAD > all groups. SBS: SZ > BP, SAD; SAD > BP. BACS Verbal memory: BP > SZ, SAD; HC > SZ, SAD, BP; SADR > BP; SZR, BPR, HC > SADR; BACS token motor: BP > SZ, SAD; HC > SZ, SAD, BP; HC, SADR > SZR, BPR; BACS digit sequencing: BP > SAD > SZ; HC > SZ, SAD, BP, SZR; BACS verbal fluency: BP > SAD > SZ; HC > SZ, SAD, BP; BACS symbol coding: BP > SZ, SAD; HC > SZ, SAD, BP, SZR, SDAR; BACS tower of London: BP > SZ, SAD; HC > SZ, SAD, BP; BACS composite Score: BP > SZ, SAD; HC > SZ, SAD, BP; SADR > BP; SZR, BPR, HC > SADR.SFS: BP, SZR, BPR, SADR, HC > SZ, SAD; BP > SAD, SZ; SZR, BPR, SADR, HC > BP; SZR, BPR > SADR; HC > BP, SAD, SZ, SZR, SADR*.

### MRI Data Acquisition and Preprocessing

Structural and functional MRI scans were acquired on 3-T scanners at each site (Scanning platforms and platforms parameters are listed in Table S1 in Supplementary Material). Structural and functional images were collected during the same scan session. Foam pads and ear plugs were used to minimize head motion and scanner noise. All the subjects were instructed to keep their eyes fixated on a crosshair, not to think about anything in particular and to move as little as possible.

### Voxel-Based Morphometry

Voxel-based morphometry analyses of structural images were performed using the VBM8 toolbox^1^ as implemented in SPM8. T1-weighted images were bias-corrected and segmented into GM, white matter (WM), and cerebrospinal fluid (CSF) by “New Segment” using a customized template, which was constructed from our 1199 study samples by DARTEL in SPM8 (40). The segmented images were normalized to Montreal Neurological Institute (MNI) space (41) using the DARTEL template and resampled to 1.5 mm × 1.5 mm × 1.5 mm voxels. In the final step, the segmented normalized images were spatially smoothed with an 8 mm × 8 mm × 8 mm full width at half maximum Gaussian kernel.

### Amplitude of Low-Frequency Fluctuations

Functional image preprocessing was carried out using data processing assistant for resting-state fMRI (DPARSF), version 2.3 (42), implemented in the MATLAB toolbox (Mathworks, Inc.). The first 9 s of each subject data were discarded before slice timing and head motion correction were performed. Then, the individual structural T1 image was coregistered to the mean functional image after motion correction. Subjects with head motion >3.0 mm of maximal translation in any direction or 3.0° of maximal rotation were excluded from further analysis. In addition, six motion parameters, CSF, and WM signals were used as nuisance covariates to reduce effects of head motion and non-neuronal BOLD fluctuations. Images were then DARTEL normalized to MNI space and resampled to 3 mm × 3 mm × 3 mm voxels. Subsequently, the time series were band-pass filtered (0.01–0.08 Hz) and linear trends removed. Then, the preprocessed time series were transformed to the frequency domain using fast Fourier transform, and power spectra obtained. Because the power of a given frequency is proportional to the square of the amplitude of this frequency component of the original time series in the time domain, the square root was calculated at each frequency of the power spectrum and the averaged square root was obtained across 0.01–0.08 Hz at each voxel. This averaged square root was taken as the ALFF. For standardization purpose, the ALFF of each voxel was divided by the global mean ALFF value. Finally, all images were spatially smoothed with an 8 mm × 8 mm × 8 mm full width at half maximum Gaussian kernel.

### Joint-Independent Component Analysis

Joint-independent component analysis is a second-level fMRI analysis method that assumes two or more features (modalities) share the same mixing matrix and maximizes the independence among joint components. ICA is performed on the horizontally concatenated feature sets (along voxels in structure–function in this case), thus uncovering patterns of data that commonly fluctuate or are connected across both modalities (see Figure S1 in Supplementary Material). It is suitable for examining a common modulation across subjects among modalities and has been applied to link a variety of feature sets in the past (43, 44). jICA (28, 45) assumes a model χ = AS where joint-independent sources (S) are linearly mixed by a common mixing parameter (A) to generate the observations data matrix (χ). In this case, we use ICA analysis algorithms to form the overall data matrix χ = [χ^GM^, χ^ALFF^] and derive spatially independent joint sources S = [S^GM^, S^ALFF^] along with their shared mixing parameter (A), which are presented as loading parameters for each subject. A total of 22 independent components were estimated according to minimum description length criteria (46) using the Group ICA of fMRI Toolbox (GIFT).^2^ The jICA was performed using the Fusion ICA Toolbox.^3^ First, the preprocessed ALFF and GM images were normalized to have the same average sum of squares to ensure that units were shared between data types. Normalization was performed on group level, so covariations among subjects were preserved. Then, the ALFF and GM data were modeled by matching the sums of squares across modalities and combined into a single data matrix, used to identify the common mixing matrix parameters using the infomax algorithm (47) shared by spatially independent joint source images (ALFF and GM images). The complete details of the method were as reported in Ref. (28).

### Statistical Analysis

A one-way analysis of variance (ANOVA) and chi-square test were carried out for demographic and clinical variables. The effects of age, sex, and site were regressed out using linear regression, and residuals of loading parameters of the independent components were compared across probands, relatives, and HC using ANOVA. Independent components showing significant main effect of group difference after Bonferroni correction were further evaluated using *post hoc* pair-wise group comparisons. A false discovery rate (FDR) correction for multiple comparisons was applied to *post hoc* tests. To assess whether the estimated ICA joint sources were associated with clinical symptoms, cognitive, or general socio-functioning, we derived associations between the residuals of the independent components and PANSS, MADRS, YMRS, BACS, and SFS scores across all available subjects. For the above association analyses, we added group as an additional covariate to covary any baseline group differences in cognition and social function.

In addition, relative risk (48) of joint structural–functional abnormalities presented by the independent components was calculated as the ratio of percentage of relatives classified as “affected” based on a threshold of 2 SD above the control mean to the percentage of healthy controls designated as “affected.” A chi-square was used to test for significance of relative risk in relatives compared with HC.

Furthermore, we used an ANCOVA model to detect the main effect of site across healthy controls and the effect of diagnosis-by-site across all groups separately. The above analyses were performed using SPSS v17.0 (Statistical Package for the Social Sciences, IBM, Chicago, IL, USA).

## Results

**Figure 1:**
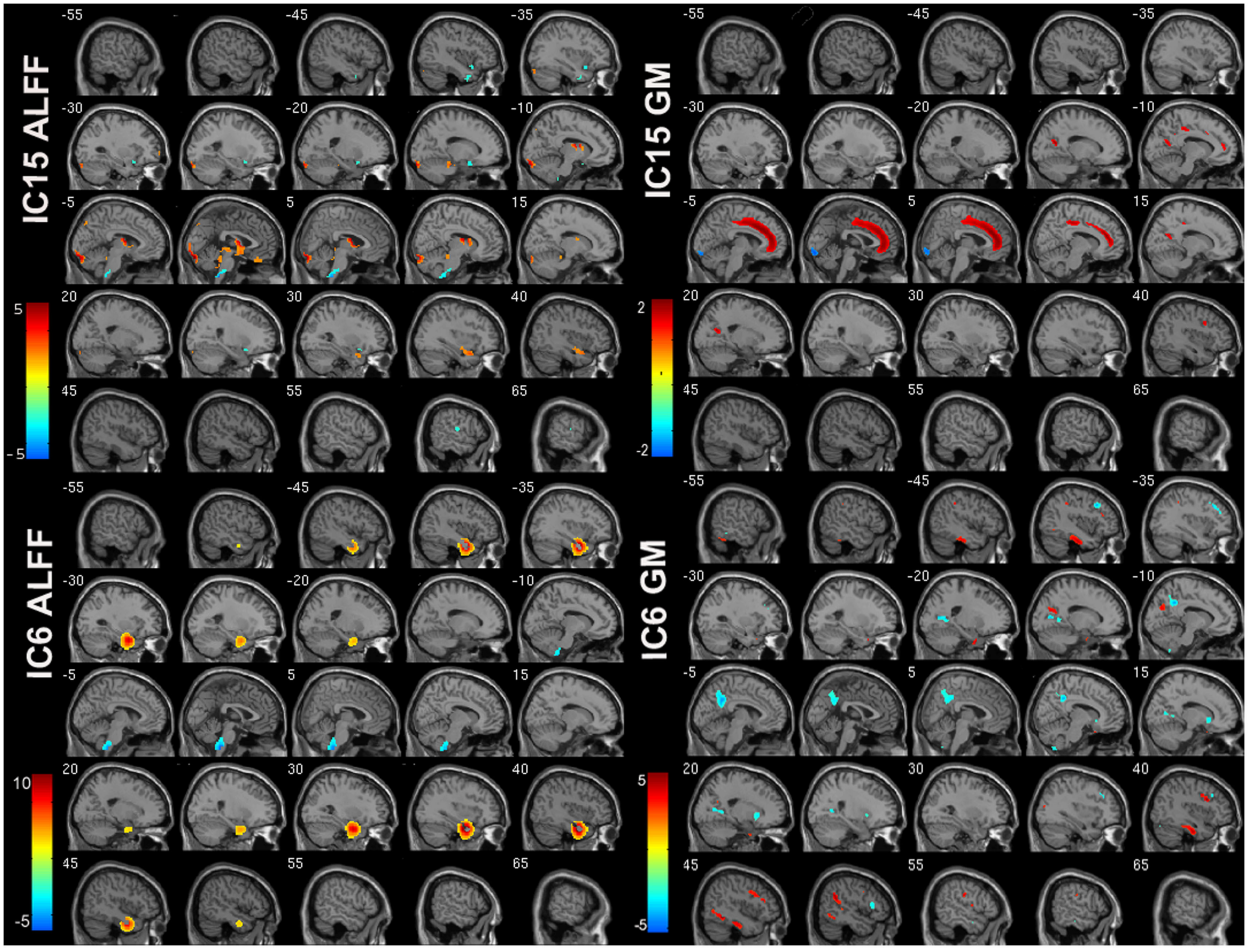
**ALFF and GM spatial maps for joint IC6 and IC15**. For display, the components were converted to *Z*-values and thresholded at |*Z*| > 2.5.

**Figure 2:**
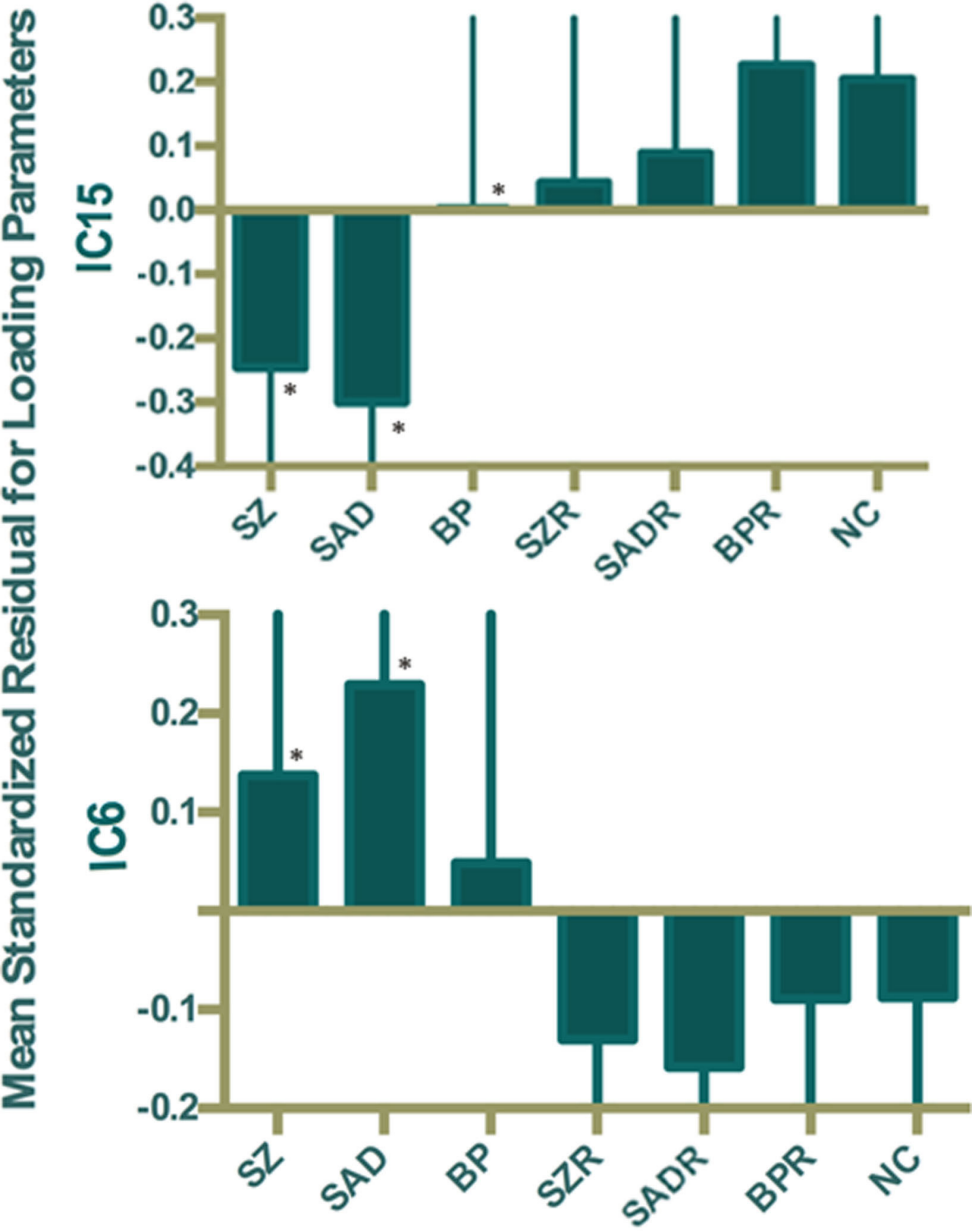
**Mean standardized residual of loading parameters for IC15 and IC6, controlling for age, sex, and site**. *Individual group had significantly different loading parameters compared to control subjects after false discovery rate (FDR) correction.

Of the 22 components estimated from the data, only network pair 6 (IC6) (*F* = 9.62; *p* = 7 × 10^−5^) and IC15 (*F* = 17.79; *p* = 2 × 10^−8^) showed a group main effects after Bonferroni correction. Talairach coordinates for the regions of IC15 and IC6 at a threshold of |*Z*| > 2.5 are summarized in Tables 2 and 3. The ALFF/GM maps of IC15 and IC6 are shown in Figure 1. As part of the output, jICA also produces loading parameters for each component pair that reflects the component’s influence at the subject level (49). This is further used to assess the between-subject differences in sMRI–fMRI association. *Post hoc* tests revealed loading parameters of IC6 were higher both in probands with SZ (*p* = 0.024) and SAD (*p* = 0.012) in relative to healthy controls. For IC 15, loading parameters were lower in probands with SZ (*p* = 0.012), SAD (*p* = 0.024), and BP (*p* = 0.036) relative to healthy controls separately. Contrasts revealed no effect of relatives and healthy controls in either IC6 or IC15. The mean standardized residual of loading parameters are shown in Figure 2. All *posthoc* measures were corrected using the FDR method (for reference).

**Table 2:** **Talairach coordinates for significant regions of component 15**.

Region	BA	Left/right volume (cc)	Left/right *Z*_max_ (*x*, *y*, *z*)
**IC15_ALFF** **CONTROLS > PROBANDS**			
Lingual gyrus	17, 18	2.3/1.4	6.9 (−3, −91, −11)/6.7 (6, −88, −11)
Thalamus	NA	1.6/2.1	4.5 (−9, −5, 11)/4.1 (6, −5, 11)
Culmen	NA	1.7/1.5	3.2 (−18, −30, −16)/3.5 (0, −39, −21)
Declive	NA	1.1/1.0	3.6 (−18, −88, −18)/3.5 (3, −79, −11)
Superior temporal gyrus	38	0.0/1.9	NA/4.0 (36, 8, −21)
Caudate	NA	0.6/1.0	5.0 (−9, 4, 14)/4.5 (9, 6, 11)
Superior frontal gyrus	6, 8, 10	1.3/0.0	2.8 (−30, 61, −3)/NA
Rectal gyrus	11	0.6/0.5	2.7 (−3, 34, −22)/2.5 (3, 37, −25)
Cuneus	17, 18, 19	0.7/0.0	3.3 (−3, −93, 0)/NA
Fusiform gyrus	18	0.6/0.0	5.0 (−21, −91, −13)/NA
Inferior occipital gyrus	17, 18	0.6/0.0	4.1 (−30, −88, −13)/NA
Precuneus	7	0.5/0.0	3.3 (−3, −76, 45)/NA
Anterior cingulate	25	0.5/0.0	3.5 (0, 2, −10)/NA
**PROBANDS > CONTROLS**			
Inferior frontal gyrus	13, 47	1.2/0.8	3.2 (−39, 11, −13)/2.8 (24, 11, −16)
Middle temporal gyrus	21, 38	1.3/0.3	3.0 (−39, 4, −30)/2.4 (48, 4, −30)
Middle occipital gyrus	18, 37	0.0/0.9	NA/2.5 (33, −76, −9)
Superior temporal gyrus	22, 38	0.6/0.0	2.6 (−39, 7, −28)/NA
Cuneus	17, 18	0.0/0.6	NA/2.6 (6, −84, 12)
Transverse temporal gyrus	41, 42	0.0/0.5	NA/3.2 (62, −14, 12)
**IC15_GM** **CONTROLS > PROBANDS**			
Cingulate gyrus	24, 31, 32	7.0/6.4	3.6 (−4, 25, 28)/3.4 (6, 28, 28)
Medial frontal gyrus	6, 8, 9, 10, 11, 32	5.9/5.6	3.2 (−7, 46, 12)/3.6 (4, 42, 15)
Anterior cingulate	10, 24, 32	4.1/4.6	3.5 (−4, 43, 13)/3.8 (7, 36, 19)
Paracentral lobule	5, 6, 31	1.1/1.0	2.9 (−3, −11, 43)/3.0 (9, −25, 44)
Precuneus	7, 31	1.3/0.6	3.0 (−13, −62, 22)/2.7 (15, −57, 24)
Posterior cingulate	30	0.8/0.7	3.2 (−13, −56, 17)/2.7 (15, −53, 15)
Middle frontal gyrus	9	0.0/0.8	NA/3.0 (40, 10, 30)
**PROBANDS > CONTROLS**			
Lingual gyrus	17, 18	1.7/1.3	3.1 (0, −86, −7)/3.0 (3, −88, −4)
Fusiform gyrus	19, 20, 37	0.3/0.7	2.3 (−52, −66, −12)/2.5 (52, −63, −13)

*NA = not applicable*.

**Table 3:** **Talairach coordinates for significant regions of component 6**.

Region	BA	Left/right volume (cc)	Left/right *Z*_max_ (*x*, *y*, *z*)
**IC6_ALFF** **PROBANDS > CONTROLS**			
Uncus	20, 28, 34, 36	5.1/4.9	8.6 (−33, −1, −28)/8.9 (33, −4, −28)
Superior temporal gyrus	38	3.4/4.4	7.8 (−33, 5, −23)/7.9 (39, 2, −20)
Middle temporal gyrus	20, 21, 38	2.6/2.9	7.6 (−39, 2, −28)/10.5 (39, −4, −28)
Parahippocampal gyrus	34, 35	2.6/2.5	8.1 (−33, −4, −20)/9.4 (36, −1, −20)
Inferior temporal gyrus	20	1.9/2.7	5.2 (−45, −4, −28)/8.4 (42, −7, −27)
Fusiform gyrus	20	0.5/0.5	4.1 (−39, −13, −25)/6.9 (42, −10, −25)
**IC6_GM** **PROBANDS > CONTROLS**			
Fusiform gyrus	20, 36, 37	1.2/1.1	3.9 (−42, −17, −24)/4.2 (42, −16, −24)
Superior temporal gyrus	13, 22, 38, 39, 41	0.8/1.0	3.3 (−27, 15, −30)/3.4 (49, −44, 19)
Middle frontal gyrus	6, 9, 10, 46	0.4/1.2	2.7 (−37, 37, 11)/3.9 (40, 13, 27)
Precuneus	7, 31	1.1/0.2	3.7 (−13, −69, 26)/2.1 (19, −60, 28)
Uncus	20, 28, 36, 38	1.0/0.3	4.1 (−19, 3, −32)/2.8 (22, 9, −27)
Inferior parietal lobule	40	0.8/0.3	3.2 (−43, −34, 39)/4.9 (46, −46, 22)
Inferior frontal gyrus	9, 47	0.3/0.8	2.5 (−39, 34, 13)/3.8 (42, 6, 33)
Inferior temporal gyrus	20, 37	0.0/0.8	NA/3.9 (40, −13, −27)
Precentral gyrus	6, 9, 13, 43	0.0/0.8	NA/3.0 (53, −4, 10)
Supramarginal gyrus	40	0.0/0.8	NA/5.0 (48, −47, 26)
Middle temporal gyrus	37, 39	0.0/0.4	NA/2.7 (34, −76, 18)
**CONTROLS > PROBANDS**			
Precuneus	7, 31	2.8/2.0	4.6 (−7, −46, 34)/3.2 (4, −48, 33)
Cingulate gyrus	31	1.8/1.6	4.2 (−7, −43, 37)/4.2 (9, −42, 37)
Middle frontal gyrus	9, 10	1.9/1.2	4.6 (−37, 22, 32)/4.5 (37, 25, 32)
Posterior cingulate	23, 30, 31	1.5/0.9	3.8 (−19, −65, 10)/3.5 (18, −64, 10)
Middle temporal gyrus	20, 21	1.7/0.5	2.8 (−56, −41, −9)/2.6 (55, −2, −20)
Lentiform nucleus	*	0.0/1.6	NA/3.2 (19, 14, −3)
Inferior frontal gyrus	45, 47	0.0/1.2	NA/3.4 (50, 22, 10)
Inferior temporal gyrus	20, 21	0.7/0.3	2.5 (−61, −21, −22)/2.6 (58, −23, −15)
Declive	*	0.0/0.7	NA/2.6 (40, −75, −15)
Cuneus	18, 23, 30	0.6/0.0	3.9 (−16, −68, 12)/NA
Precentral gyrus	6, 9	0.3/0.3	5.2 (−37, 21, 36)/3.0 (50, 19, 7)
Lingual gyrus	18, 19	0.4/0.2	3.1 (−16, −51, 5)/2.5 (22, −54, 5)
Parahippocampal gyrus	30	0.3/0.2	2.7 (−22, −52, 5)/3.4 (19, −49, 5)

We correlated the loading parameters of both ICs with the PANSS, MADRS, YMRS, BACS, SBS, and SFS scores for all available subjects. While, loading parameters of IC6 showed no correlation with any of the above scores. Loading parameters of IC15 were positively correlated with the BACS composite scores, BACS symbol coding scores, and BACS tower of London scores after FDR correction (Table 4).

**Table 4:** **Significant correlations between IC15 loading parameters and the brief assessment of cognition in schizophrenia adjusted using FDR correction for multiple comparisons**.

IC15	BACS (*z*)	*R*	p*
	Symbol coding	0.175	0.001
	Tower of London	0.157	0.003
	Composite score	0.169	0.001

*FDR corrected.

There was no significant difference between relatives and healthy controls in relative risk of joint structural–functional abnormalities. Main effects of site were observed for both IC6 (*F* = 40.63, *p* = 4 × 10^−30^) and IC15 (*F* = 9.98, *p* = 1 × 10^−8^). However, more importantly, no diagnosis-by-site interaction was noted.

## Discussion

The human brain is connected on a variety of different spatial scales, from synaptic signaling at the cellular level to a more broad systems level containing inter-regional communication across physically distant brain regions. A natural starting point to examine system level architecture is by using traditional “unimodal” techniques. However, it is also possible to apply more advanced statistical techniques to define “cross-modal” brain relationships between disparate measures (e.g., structure and function). In this study, we took this approach to detect common and unique abnormalities in a large psychosis sample by fusing two modalities (rs-fMRI and sMRI) using a jICA approach across the psychotic spectrum (SZ, SAD, and psychotic BP) and to investigate which of those are shared by their unaffected first-degree relatives, suggesting possible endophenotypes. Since multimodal techniques such as joint ICA capture “cross-feature” information simultaneously, they naturally contribute to a different set of information compared to their individual “unimodal” counterparts.

By using a jICA approach, two components showing group differences were identified. Joint loadings computed from IC15 showed significant differences in SZ, SAD, and BP (compared to HC), while IC6 distinguished only SZ and SAD from controls. However, first-degree, non-psychotic relatives showed no common abnormalities with the probands and no significant differences compared to HC. Consistent with this finding, we found that the relative risk estimates for the two components were non-significant. Brain changes in function and structure correlated with certain sub-scales of the BACS inventory, suggesting direction relationships between affected structure–function patterns and cognitive function scores.

The IC15-ALFF network encompassed regions, including thalamus, cerebellum, prefrontal cortex, and caudate, which are primarily involved in the prefrontal–striatal–thalamic–cerebellar network that has been implicated in the pathophysiology of both SZ (50, 51) and BP (52), supported by growing evidence (53–59). The prefrontal cortex plays a critical role in executive cognitive control, whereas the striatum and the cerebellum, which are connected with the prefrontal cortex via thalamus (60, 61), are also involved in executive function, working memory, spatial cognition, and language (62). The thalamus not only functions as a nexus to integrate cortical and subcortical activity (63, 64) but is also implicated in processing and integrating sensory information via connections with sensory–motor cortices (65, 66). Remaining brain regions in IC15-ALFF included visual areas, such as lingual gyrus, cuneus, fusiform gyrus, and occipital cortex; and auditory-related areas, such as superior temporal and transverse temporal gyri, which may indicate dysconnectivity between sensory cortices and thalamus in psychosis. Thus, dysfunctions in this network may be associated with abnormal cognition, difficulty in coordinating processing, prioritization, retrieval, and expression of the information associated with psychotic symptoms (51, 67). Consistent with our results, Anticevic et al. (68) documented that thalamic connectivity with prefrontal–striatal–cerebellar regions successfully classified SZ and psychotic bipolar patients, suggesting that this network may be abnormal across diagnoses. Interestingly, IC15 also showed decreased GM in regions that constitute the functional DMN (69), consistent with previous findings in both SZ/SAD and BP that showed reduced GM (24, 70–75). Two regions within IC15 showing increased GM in psychosis probands were lingual gyrus and fusiform gyrus, consistent with previous studies with larger fusiform gyrus in both SZ and BP (76) and larger lingual gyrus in SZ (77) compared with healthy controls. Positive correlation between the loading parameters of IC15 and BACS scores support a link between abnormalities in prefrontal–striatal–thalamic–cerebellar network and DMN and cognitive function, consistent with previous studies (38, 78–84). Interestingly, symbol coding was the most sensitive indicator of genetic liability for SZ/SAD and performance distinguished psychotic BP from major depression (85).

Among the IC6-ALFF regions, a subset of temporal regions showed higher ALFF in both SZ and SAD in relative to HC and no difference between BP and controls. These results are consistent with previous studies that increased ALFF were found in inferior temporal gyrus, uncus, fusiform gyrus (86), superior temporal gyrus (87), and parahippocampal gyrus (88) in SZ when compared to HC. All these regions overlap with the IC6-GM regions, which suggest that both temporal lobe function and structure are disturbed in SZ and SAD (89, 90). Regions noted as part of IC6-GM have been consistently shown to have reduced GM in SZ compared to HC (91, 92), often in association with psychotic symptoms (93). Another jICA study (28) that combined fMRI (from an auditory oddball task) and sMRI data showed similar brain regions to ours with both increased GM and abnormal activation primarily located in temporal lobe in SZ compared to HC. Furthermore, consistent with our results, Calhoun et al. (94) reported that temporal lobe functional data successfully discriminated HC from SZ during an auditory oddball task, and combined temporal lobe and DMN data in resting-state discriminated between SZ and psychotic BP, consistent with well-studied temporal lobe anomalies in SZ (45).

A natural question is what mechanisms may be responsible for these types of long-distance structural–functional couplings and how functional data in one region might be linked to a different/remotely located functional region or vice versa. One possibility is that the local GM volume in one region affects the quantity of functional output from that region, which in turn has a causal influence on synaptic input arriving at a distal cortical location, thus suggesting a relationship from structure to downstream functional response. Alternatively, the causal direction of the relationship could be reversed, with the amount of functional activity in a region influencing the structural volume of a downstream region, either through excitotoxic or neurotrophic influences. Notably, an excitotoxic downstream effect would be a mechanism by which increased functional activity in one region (if consistently elevated) could lead to decreased structural volume in another region. In all these hypothesized mechanisms, it is possible that the observed structure–function relationship is mediated by intervening regions (either direct or indirect connections). Overall, multiple mechanisms and pathways could lead to a coupling between structural and functional characteristics of the brain. Because these mechanisms are not static, it seems likely that the strength and directionality of structure–function correlations could vary in psychiatric populations, such as SZ, where brain connectivity is compromised in general.

The current subject sample largely overlapped with two previously published studies from our group investigating sMRI and ALFF individually in a more traditional “unimodal” voxel-wise fashion (24, 95). Importantly, these previous studies did not look at connectivity of regions as being presently reported here. Overall, in these previously reported studies, we identified larger structural and functional anomalies in SZ/SAD compared to PBP, consistent with findings from the current jICA approach. However, not all regions reported in the current study overlapped with previous findings. This is not surprising, given that jICA (a) is a purely data-driven (blind) technique, (b) captures data patterns that are linked between multimodal features, and (c) explores connectivity patterns among large-scale networks as opposed to regional effects. The jICA approach is therefore a completely different and novel approach to explore the data compared to traditional voxel-wise/regional methods, as reported previously.

Advantages of our study are the relatively large population with psychosis probands and relatives across the psychosis dimension and we combine two different data type to investigate the abnormalities. Limitations of the study include the potential confounds related to medication and illness state (96).

In summary, this study provides evidence based on a large sample of psychosis probands and relatives that associate between functional brain alterations (ALFF) in a prefrontal–striatal–thalamic–cerebellar network and structural abnormalities (GM) in DMN are common disturbances across psychotic diagnoses, while the alteration in both function and structure in temporal lobe are unique to SZ and SAD. Our results also suggest that SAD is more similar to SZ across the schizo-bipolar spectrum. Comparisons between relatives and HC reveal that these structural–functional abnormalities may be psychosis biomarkers rather than endophenotypes. Future research investigating associations between different data types, fusing information from different modalities, may provide more clues to uncover the mechanisms of psychotic illnesses, leading to a biologically driven classification and more efficient and ultimately personalized treatment strategies.

## Conflict of Interest Statement

The authors declare that the research was conducted in the absence of any commercial or financial relationships that could be construed as a potential conflict of interest.

## Footnotes

^1^http://dbm.neuro.uni-jena.de/vbm

^2^http://mialab.mrn.org/software/gift, version 2.0a

^3^http://mialab.mrn.org/software/fit, version 1.2c
