# Supplementary material for: Large-Scale Fusion of Gray Matter and Resting-State Functional MRI Reveals Common and Distinct Biological Markers across the Psychosis Spectrum in the B-SNIP Cohort
Source: Front Psychiatry. 2015 Dec 21;6:174. doi: 10.3389/fpsyt.2015.00174 (PMC4685049; doi:10.3389/fpsyt.2015.00174)
Supplement: Supplementary file 1 [file Table_1.doc]

Supplementary TABLE 1. Scanner parameters across all sites.

| **fMRI** | **TR (ms)** | **TE (ms)** | **Flip angle (degree)** | **Slices (N)** | **Matrix (mm)** | **Voxel Size (mm)** | **Vendor/Model** |
| --- | --- | --- | --- | --- | --- | --- | --- |
| **Baltimore** | 2210 | 30 | 70 | 36 | 64x64 | 3.4x3.4x3 | Siemens TrioTim |
| **Boston** | 3000 | 27 | 60 | 30 | 64x64 | 3.4x3.4x5 | GE Signa HDX |
| **Chicago** | 1775 | 27 | 60 | 29 | 64x64 | 3.4x3.4x4 | GE Signa HDX |
| **Dallas** | 1500 | 27 | 60 | 29 | 64x64 | 3.4x3.4x4 | Philips |
| **Detroit** | 1570 | 22 | 60 | 29 | 64x64 | 3.4x3.4x4 | Siemens TrioTim |
| **Hartford** | 1500 | 27 | 70 | 29 | 64x64 | 3.4x3.4x5 | Siemens Allegra |
| **sMRI** | **TR (ms)** | **TE (ms)** | **Flip angle (degree)** | **Slices (N)** | **Matrix (mm)** | **Voxel Size (mm)** | **Vendor** |
| **Baltimore** | 2300 | 2.91 | 9 | 160 | 256x240 | 1x1x1.2 | Siemens TrioTim |
| **Boston** | 6.98 | 2.84 | 8 | 166 | 256x256 | 1x1x1.2 | GE Signa HDX |
| **Chicago** | 6.98 | 2.84 | 8 | 166 | 256x256 | 1x1x1.2 | GE Signa HDX |
| **Detroit** | 2300 | 2.94 | 9 | 160 | 256x240 | 1x1x1.2 | Siemens TrioTim |
| **Dallas** | 6.6 | 2.8 | 8 | 170 | 256x256 | 1x1x1.2 | Philips |
| **Hartford** | 2300 | 2.91 | 9 | 160 | 256x240 | 1x1x1.2 | Siemens Allegra |
