# Supplementary material for: Large-Scale Fusion of Gray Matter and Resting-State Functional MRI Reveals Common and Distinct Biological Markers across the Psychosis Spectrum in the B-SNIP Cohort
Source: Front Psychiatry. 2015 Dec 21;6:174. doi: 10.3389/fpsyt.2015.00174 (PMC4685049; doi:10.3389/fpsyt.2015.00174)
Supplement: Supplementary file 2 [file Table_2.docx]

|  | **Schizophrenia Probands**  **(n=220)** | **Schizoaffective Disorder Probands**  **(n=147)** | **Psychotic Bipolar probands**  **(n=180)** | **Relatives of schizophrenia**  **(n=150)** | **Relatives of schizoaffective disorder**  **(n=126)** | **Relatives of Psychotic Bipolar (n=134)** | **Health Control**  **(n=242)** |
| --- | --- | --- | --- | --- | --- | --- | --- |
|  |  |  |  |  |  |  |  |
|  |  |  |  |  |  |  |  |
| **Unknown Medication History, (n) %** | 5 (2.3) | 0 (0.0) | 4 (2.2) | 3 (2.0) | 5 (4.0) | 2 (1.5) | 3 (1.24) |
|  |  |  |  |  |  |  |  |
| **Medication data for subjects with medication history reported** | **n=215** | **n=147** | **n=176** | **n=147** | **n=121** | **n=132** | **n=239** |
| **No Medication taken, (n) %** | 6 (2.8) | 8 (5.4) | 8 (4.6) | 56 (38.1) | 34 (28.1) | 42 (31.8) | 115 (48.1) |
| **Not on Psychotropic Medications, (n) %** | 16 (7.4) | 11 (7.5) | 14 (8.0) | 121 (82.3) | 91 (75.2) | 91 (68.9) | 229 (95.8) |
| **On >1 Psychotropic Medication, (n) %** | 148 (68.8) | 125 (85.0) | 137 (77.8) | 8 (5.4) | 10 (8.3) | 16 (12.1) | 3 (1.3) |
|  |  |  |  |  |  |  |  |
| **Antipsychotic (Any), (n) %** | 193 (89.8) | 125 (85.0) | 124 (70.5) | 0 (0.0) | 0 (0.0) | 0 (0.0) | 0 (0.0) |
| **A. First Generation** | 25 (11.6) | 12 (8.2) | 12 (6.8) | 0 (0.0) | 0 (0.0) | 0 (0.0) | 0 (0.0) |
| **B. Second Generation** | 168 (78.1) | 112 (76.2) | 112 (63.6) | 0 (0.0) | 0 (0.0) | 0 (0.0) | 0 (0.0) |
| **Mood Stabilizer (Any), (n) %** | 47 (21.9) | 82 (55.8) | 122 (69.3) | 1 (0.7) | 7 (5.8) | 6 (4.6) | 0 (0.0) |
| **A. Lithium** | 15 (7.0) | 21 (14.3) | 43 (24.4) | 0 (0.0) | 0 (0.0) | 4 (3.0) | 0 (0.0) |
| **B. Anticonvulsants** | 32 (14.9) | 61 (41.5) | 79 (44.9) | 1 (0.7) | 7 (5.8) | 2 (1.5) | 0 (0.0) |
| **Antidepressant (Any), (n) %** | 83 (38.6) | 82 (55.8) | 82 (46.6) | 17 (11.6) | 19 (15.7) | 32 (24.2) | 4 (1.7) |
| **A. SSRIs/SNRIs** | 55 (25.6) | 47 (32.0) | 40 (22.7) | 16 (10.9) | 14 (11.6) | 24 (18.2) | 3 (1.3) |
| **B. Tricyclic** | 2 (0.9) | 2 (1.4) | 5 (2.8) | 0 (0.0) | 3 (2.5) | 2 (1.5) | 0 (0.0) |
| **C. MAO Inhibitors** | 0 (0.0) | 0 (0.0) | 0 (0.0) | 0 (0.0) | 0 (0.0) | 0 (0.0) | 0 (0.0) |
| **D. Miscellaneous** | 26 (12.1) | 33 (22.5) | 37 (21.0) | 1 (0.7) | 2 (1.7) | 6 (4.6) | 1 (0.4) |
| **Anxiolytic/Sedatives/Hypnotic, (n) %** | 48 (22.3) | 48 (32.7) | 55 (31.3) | 12 (8.2) | 9 (7.4) | 11 (8.3) | 6 (2.5) |
| **Anticholinergic/Antiparkinsonian, (n) %** | 37 (17.2) | 18 (12.2) | 15 (8.5) | 0 (0.0) | 0 (0.0) | 0 (0.0) | 0 (0.0) |
| **Stimulants, (n) %** | 8 (3.7) | 8 (5.4) | 18 (10.2) | 1 (0.7) | 4 (3.3) | 5 (3.8) | 1 (0.4) |
| **Miscellaneous, Psychotropic/Centrally Active, (n) %** | 5 (2.3) | 5 (3.4) | 5 (2.8) | 0 (0.0) | 1 (0.8) | 0 (0.0) | 0 (0.0) |

Supplementary TABLE 2. Mediation data for the study sample.
