# Supplementary figures and images for: Large-Scale Fusion of Gray Matter and Resting-State Functional MRI Reveals Common and Distinct Biological Markers across the Psychosis Spectrum in the B-SNIP Cohort
Source: Front Psychiatry. 2015 Dec 21;6:174. doi: 10.3389/fpsyt.2015.00174 (PMC4685049; doi:10.3389/fpsyt.2015.00174)

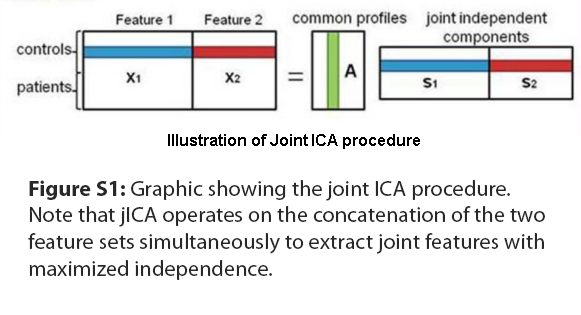

Supplement: Supplementary file 3 [file Image_1.tif]

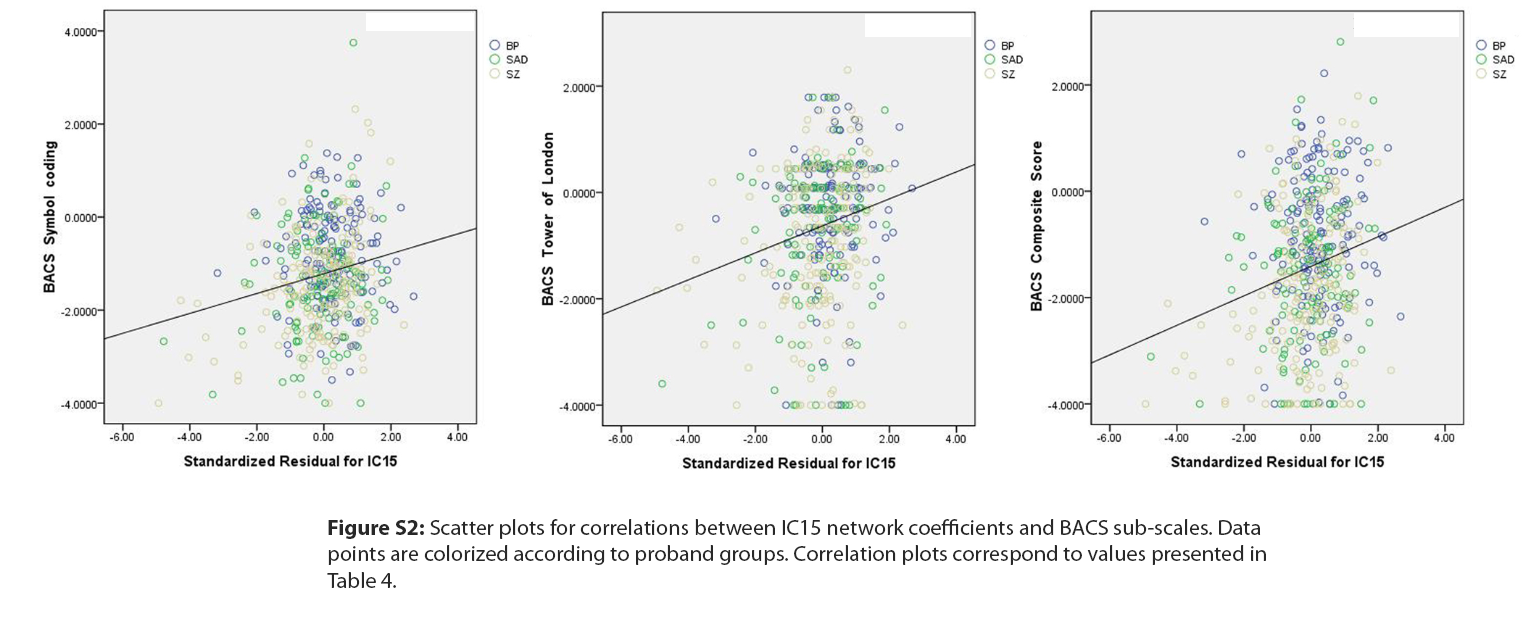

Supplement: Supplementary file 4 [file Image_2.tif]
